# Cognitive Impairment and Synaptic Dysfunction in Cardiovascular Disorders: The New Frontiers of the Heart–Brain Axis

**DOI:** 10.3390/biomedicines12102387

**Published:** 2024-10-18

**Authors:** Teresa Soda, Teresa Pasqua, Giovambattista De Sarro, Francesco Moccia

**Affiliations:** 1 Department of Health Sciences, University of Magna Graecia, 88100 Catanzaro, Italy; teresa.pasqua@unicz.it (T.P.); desarro@unicz.it (G.D.S.); 2 Department of Medicine and Health Sciences “V. Tiberio“, University of Molise, 86100 Campobasso, Italy; francesco.moccia@unimol.it

**Keywords:** heart–brain axis, cardiovascular disorders, synaptic plasticity, cardiogenic dementia, cognitive impairment, heart failure, metabolic syndrome, arrhythmias, NMDA receptors, long-term potentiation

## Abstract

Within the central nervous system, synaptic plasticity, fundamental to processes like learning and memory, is largely driven by activity-dependent changes in synaptic strength. This plasticity often manifests as long-term potentiation (LTP) and long-term depression (LTD), which are bidirectional modulations of synaptic efficacy. Strong epidemiological and experimental evidence show that the heart–brain axis could be severely compromised by both neurological and cardiovascular disorders. Particularly, cardiovascular disorders, such as heart failure, hypertension, obesity, diabetes and insulin resistance, and arrhythmias, may lead to cognitive impairment, a condition known as cardiogenic dementia. Herein, we review the available knowledge on the synaptic and molecular mechanisms by which cardiogenic dementia may arise and describe how LTP and/or LTD induction and maintenance may be compromised in the CA1 region of the hippocampus by heart failure, metabolic syndrome, and arrhythmias. We also discuss the emerging evidence that endothelial dysfunction may contribute to directly altering hippocampal LTP by impairing the synaptically induced activation of the endothelial nitric oxide synthase. A better understanding of how CV disorders impact on the proper function of central synapses will shed novel light on the molecular underpinnings of cardiogenic dementia, thereby providing a new perspective for more specific pharmacological treatments.

## 1. Introduction

The heart–brain axis (HBA) is based upon the bidirectional flow of information between the heart and the brain, which becomes evident when the dysfunction in one system leads to a significant impairment in the function (and even in the structure) of the other [1,2,3]. The functional interplay between the cardiovascular (CV) and nervous systems has shaped the concept of neurocardiology, a branch of medicine that aims at investigating the pathological and therapeutic implications of the HBA [4]. The HBA is perhaps more known to cardiologists rather than neurologists as the potential ability of the central nervous system (CNS) to cause CV disorders through the cardiomotor sympathetic and parasympathetic outflow of the autonomous nervous system has long been recognized [1,4]. An imbalance between the sympathetic (cardioexcitatory) and parasympathetic (cardioinhibitory) tone could lead to emotional stress-induced cardiomyopathy syndromes, including Takotsubo syndrome and neurogenic stunned myocardium [1,4,5]. In addition, the heart can promote catecholamine secretion via the hypothalamic–pituitary–adrenal (HPA) axis, which also promote cortisol release from the adrenal glands. The neuroendocrine storm triggered by the HPA further contributes to myocardial injury by predisposing the heart to insults, such as ischemia, inflammation, and ionic disturbances [6,7]. The autonomic imbalance induced by emotional and/or physical stress could also result in hypertension, increased afterload, and, ultimately, congestive heart failure (HF) by impairing endothelial signaling, mean arterial pressure, cardiac phenotype and function (e.g., left ventricular hypertrophy and arrhythmia) and the renin–angiotensin–aldosterone system [5,8]. The harmful consequences of the increased sympathetic tone on the CV system have gained further momentum during the recent COronaVIrus Disease 19 (COVID-19) pandemic, in which the HBA dysfunction has contributed to worsening the outcome of COVID-19 patients [9].

**Figure 1:**
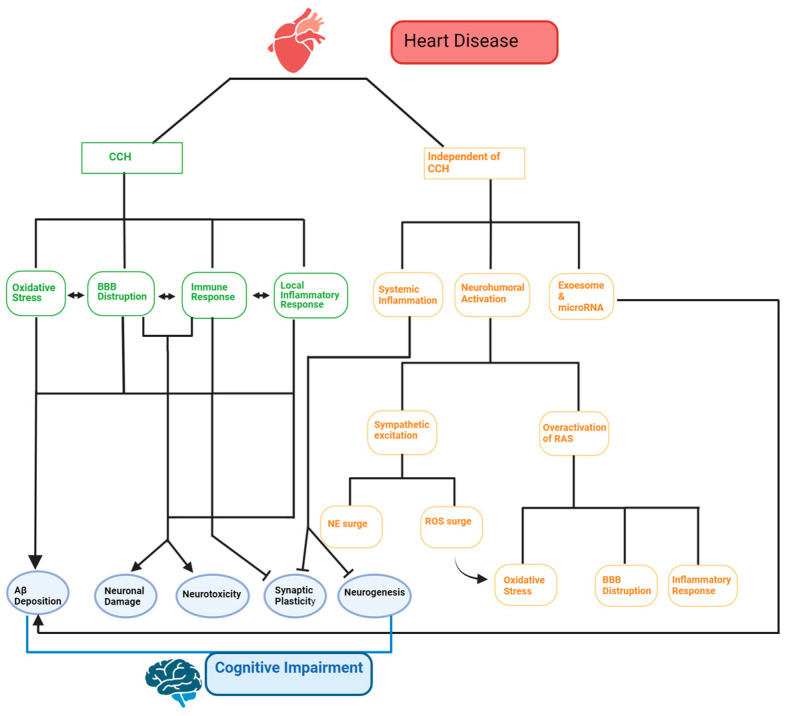
Cardiogenic dementia: a multifaceted pathway. Cardiogenic dementia can result from cognitive impairment following heart disease. This impairment can occur due to chronic cerebral hypoperfusion (CCH) or independently of it. CCH arises when long-term heart damage reduces cardiac output. This triggers a chain of events involving oxidative stress, local inflammation, immune responses, and blood–brain barrier (BBB) disruption. Cardiogenic dementia can also occur without changes in cerebral blood flow (CBF). This involves systemic inflammation, neurohumoral activation, and the release of exosomes. Increased norepinephrine (NE) and reactive oxygen species (ROS) can result from sympathetic excitation. Additionally, the overactivation of the renin–angiotensin system (RAS) leads to oxidative stress, BBB disruption, and inflammation. In conclusion, heart disease can contribute to amyloid-beta protein (Aβ) deposition, neuronal damage, and neurotoxicity. It can also hinder synaptic plasticity and neurogenesis through both CCH-dependent and -independent mechanisms. These factors collectively worsen cognitive function. Made with BioRender.

However, CV diseases, pivotal among which are HF, metabolic syndrome, and arrhythmias, may also result in severe neurological disorders as a consequence of cerebral hypoperfusion and cardioembolic stroke or, in the presence of myocardial injury, because of systemic inflammation and neurohumoral activation [2,10,11,12,13]. The ability of the CV system to regulate neuronal signaling and synaptic plasticity may lead patients with cardiac-related risk factor to present severe cognitive deficits [2,12], a condition for which the term “cardiogenic dementia” has been coined (Figure 1) [2,14]. The mechanisms by which CV dysfunction may lead to cognitive defects and induce cardiogenic dementia have been widely investigated and discussed in an excellent recent review article [2]. Nevertheless, the pathophysiological link between CV disorders and the impairment of neuronal signaling and synaptic plasticity, which underlies cardiogenic dementia at molecular/cellular levels, remains still elusive. Herein, we first describe the ionic mechanisms of learning and memory by focusing our attention on the excitatory glutamatergic synapses, which can experience an increase or a decrease in the strength of synaptic transmission that underlie cognitive and emotional processes. Then, we illustrate how HF, metabolic syndrome, including hypertension, obesity, diabetes and insulin resistance, and endothelial dysfunction, and arrhythmias may impair the ionic and synaptic mechanisms responsible for synaptic plasticity in the CNS. A better understanding of how CV disorders impact on the proper function (and structure) of central synapses is not only expected to shed novel light on the molecular underpinnings of cardiogenic dementia but also to provide a new prospect for more effective pharmacological treatments.

## 2. The Ionic Mechanisms of Learning and Memory: The Unexpected Targets of Cardiovascular Disorders

Synaptic plasticity, the ability of synapses to dynamically strengthen or weaken in response to neural activity, is a fundamental mechanism underpinning learning and memory. Therefore, cardiogenic dementia primarily affects the signaling pathways that underpin memory formation consolidation and shape emotional behavior at excitatory synapses in the hippocampus and other brain regions, such as the amygdala and prefrontal cortex (PFC). Excitatory neurotransmission in the brain is mediated by glutamate, which is released from pre-synaptic terminals and targets both ionotropic and metabotropic receptors on the postsynaptic neurons. CV diseases severely interfere with glutamatergic signaling and with the downstream pathways that lead to memory and learning and shape emotional and behavioral skills. 

**Figure 2:**
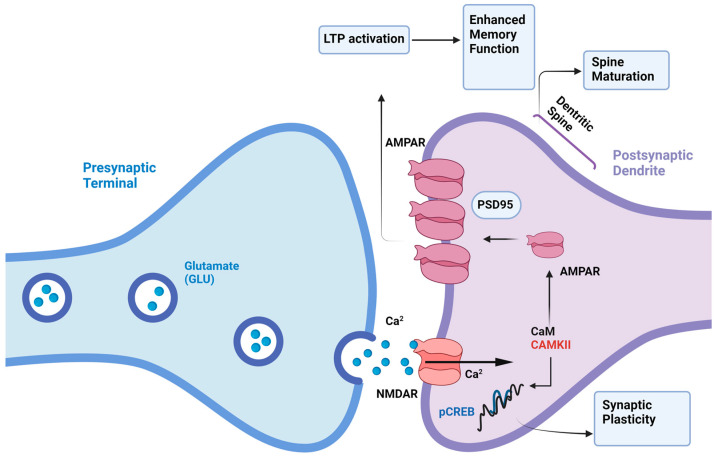
Molecular mechanisms of LTP in the hippocampus. The molecular mechanisms underlying LTP, involving signaling pathways mediated by N-methyl-D-aspartate (NMDA) receptors (NMDARs) and calcium ions (Ca^2+^). At excitatory synapses, synaptic plasticity is primarily mediated by alterations in postsynaptic ionotropic glutamate receptors, particularly α-amino-3-hydroxy-5-methyl-4-isoxazolepropionic acid (AMPA) receptors (AMPARs). NMDARs play a crucial role in initiating LTP by acting as coincidence detectors for pre- and postsynaptic firing patterns. An NMDAR-mediated rise in postsynaptic Ca^2+^ activates the Ca^2+^/Calmodulin (CaM)-dependent protein-kinase II (CaMKII). CaMKII-dependent phosphorylation, in turn, drives AMPAR incorporation to postsynaptic density in a post-synaptic density protein 95 (PSD-95)-dependent manner. Furthermore, CaMKII may phosphorylate cAMP response element-binding protein (CREB), the transcription factor regulating the expression of postsynaptic proteins and driving the physical expansion of dendritic spines. Made with BioRender.

Long-term potentiation (LTP) and long-term depression (LTD) are two well-characterized forms of synaptic plasticity. LTP, characterized by a persistent increase in synaptic strength, is often associated with the acquisition of new information. Conversely, LTD, a long-lasting decrease in synaptic strength, is thought to be involved in processes such as forgetting and the refinement of neural circuits. While the hippocampus has been the primary model system for studying synaptic plasticity, recent evidence suggests that these mechanisms are widespread throughout the brain [15,16,17,18,19]. The molecular mechanisms underlying LTP and LTD are complex and involve a variety of signaling pathways, including those mediated by N-methyl-D-aspartate (NMDA) receptors (NMDARs), calcium ions (Ca^2+^), and second messenger systems (Figure 2) [17,20,21,22,23,24,25]. At excitatory synapses, synaptic plasticity is primarily mediated by alterations in the function and number of postsynaptic ionotropic glutamate receptors, particularly α-amino-3-hydroxy-5-methyl-4-isoxasolepropionic acid (AMPA) receptors (AMPARs), kainate receptors, and NMDARs (Figure 2) [26,27]. Given AMPARs’ dominant role in basal synaptic transmission, much research on LTP and LTD mechanisms has focused on understanding how AMPAR-mediated synaptic responses are modulated [17,26,27]. A prevailing theory posits that NMDARs play a crucial role in initiating various forms of activity-dependent LTP and LTD by acting as a coincidence detector for pre- and postsynaptic firing patterns [15]. This property depends on the Mg^2+^-dependent inhibition of the NMDAR channel at resting membrane potential and their high permeability to Ca^2+^. An NMDAR-mediated rise in postsynaptic Ca^2+^ activates kinases, notably Ca^2+^/Calmodulin-dependent protein kinase II (CaMKII), protein kinase A (PKA), protein kinase C (PKC), and mitogen-activated protein kinase (MAPK), and protein phosphatases, such as calcineurin, ultimately results in an increase (Figure 2) or decrease in AMPAR density and/or conductance [17,28,29].

While synaptic AMPARs have long been recognized as undergoing long-lasting modulation by synaptic activity, the question of whether synaptic NMDARs exhibit similar plasticity has been a subject of intense interest. Once thought to be relatively stable at synapses, synaptic NMDARs are now emerging as dynamic and capable of activity-dependent modulation, akin to AMPARs. Our understanding of the mechanisms of LTP of NMDAR-mediated excitatory postsynaptic potentials (NMDAR-EPSCs) has advanced significantly [30,31,32,33,34,35]. These studies converged on the notion that NMDAR-EPSCs may also undergo synaptic potentiation upon an increase in dendritic Ca^2+^ levels that is shaped by both NMDARs and group 1 metabotropic glutamate receptors (mGluR1 and mGluR5) [31,34,35,36]. In contrast to LTP, LTD of NMDAR-mediated synaptic responses has been consistently observed in response to induction protocols that elicit NMDAR-dependent LTD of AMPAR responses [37,38,39,40]. Therefore, just like synaptic AMPARs, synaptic NMDARs can also be bidirectionally modified by different patterns of synaptic activity. There is a wealth of evidence, not addressed here, that NMDARs are tightly regulated by experience during development. Our understanding of the mechanisms of activity-dependent synaptic plasticity of NMDA receptors is only beginning to emerge.

A more nuanced understanding of the temporal dynamics of synaptic plasticity has been provided by the discovery of spike timing-dependent plasticity (STDP)**.** STDP reveals that the precise timing of pre- and postsynaptic spikes determines whether a synapse will strengthen or weaken. This bidirectional nature of STDP suggests a Hebbian-like learning rule, where synapses are strengthened when they are active during the generation of a postsynaptic spike and weakened when they are active but do not contribute to the generation of a postsynaptic spike. Recent studies have highlighted the role of various molecular mechanisms, including NMDARs, Ca^2+^ signaling, and protein synthesis, in regulating both LTP and LTD in canonical STDP [18,41,42].

Consistent with their critical role in learning and memory, as well as in socio-emotional and behavioral skills, LTP and LTD induction and maintenance are severely compromised in neurodegenerative and neuropsychiatric disorders, such as Alzheimer’s disease, Parkinson’s disease, dementia, schizophrenia, depression, and autism spectrum disorders [43,44,45,46,47]. Therefore, it is not surprising that cardiogenic dementia may also target the molecular mechanisms that strengthen or weaken synaptic transmission at central glutamatergic synapses, thereby leading the patients to cognitive impairment.

## 3. Heart Failure and Cognitive Impairment

HF is a complex clinical syndrome characterized by the heart’s inability to pump and/or refill with blood, thereby resulting in a reduced cardiac output (HF with reduced ejection fraction or HFrEF) or in an adequate cardiac output secondary to an increase in the left ventricular filling pressure due to robust neurohormonal activation (HF with preserved ejection fraction, HFpEF) [48]. HF has been estimated to affect 30–50 million patients worldwide, but its incidence is expected to rise as a consequence of the longer global average life expectancy and the availability of novel medications that substantially improve survival after HF diagnosis [49]. While the CV implications of HF are well documented, its effects on cognitive function and synaptic integrity are increasingly recognized as critical components of the disease [12,50]. Intriguingly, HF increases the risk of developing cognitive impairment, including deficits in learning and working memory, more than four-fold as compared to healthy control subjects [51]. In addition, HF patients are comorbid with anxiety and depression, which contribute to further worsening their quality of life and self-care [52]. This relationship underscores the necessity of understanding the molecular mechanisms that link HF to synaptic dysfunction. HF may affect cognitive function by reducing cerebral blood flow (CBF) [53,54], thereby resulting in hypoperfusion and/or hypoxia, and by triggering a strong neuroinflammatory axis [55,56]. These, in turn, lead to significant loss of cerebral grey matter loss and damage in brain regions that are involved in memory and emotions, such as the hippocampus, amygdala, and PFC [57,58]. However, the neural substrates and molecular mechanisms that contribute to HF-induced cognitive and emotional decline are still unclear. The brain receives ≈20% of the cardiac output [59], thereby consuming ≈60% of the energy-produced oxygen to maintain the neuronal resting potential, support neuronal firing, and enable synaptic transmission [60]. In accordance with this notion, it has long been known that persistent synaptic failure may result from mild or moderate cerebral hypoxia due to ATP depletion and down-regulation of AMPARs and NMDARs as well as of many proteins involved in synaptic vesicle trafficking, including Syntaxin-1A, Synaptogyrin-1, and SV-2 [61,62,63,64]. However, the incidence of cognitive decline does not differ significantly between HF patients with preserved versus reduced ejection fraction. The prevalence of stroke is also similar in HFrEF and HFpEV patients [55]. Therefore, additional mechanisms must contribute to impair synaptic function and favor cognitive and emotional decline in HF.

A recent investigation unveiled that neuroinflammation secondary to HF may impair synaptic function and plasticity, particularly in the dorsal hippocampus (DH) [65], which plays a pivotal role in memory and learning [16,25]. By using an ischemic HF rat model, Althammer and colleagues first confirmed that microglia, the resident immune cells of the CNS, undergo a transition towards a pro-inflammatory phenotype in DH, which is progressive in time and depends on the severity of HF [65]. Then, they found an increase in the inflammatory signal, including IL (interleukin) 1β (IL-1β), tumor necrosis factor-α (TNF-α), and C1q, which was restricted to DH and was associated with an astrocytic shift from a neuroprotective to a neurotoxic condition. In accordance with these findings, the CA1 region of HF rats showed an increase apoptotic rate and a decreased excitability of pyramidal neurons, as shown by their depolarized resting potential and reduced input/output relationship [65]. Therefore, HF reduces the ability of CA1 pyramidal neurons to properly process the incoming information by firing a train of action potential at the proper rate. Furthermore, this investigation provided additional evidence in support of the pathogenic role of the renin–angiotensin system in HF-induced neuroinflammation [56]. Angiotensin II (Ang II) is a pro-inflammatory peptide that is released in circulation after AMI [56] and may target AT1a receptors (AT1aRs) in multiple brain cells, including neurons, astrocytes, microglia, and oligodendrocytes [66]. The blood–brain barrier (BBB) was found to be leaky in the DH of HF rats, thereby potentially enabling circulating Ang II to cross the BBB and trigger the local cascade of neuroinflammation [65]. In accordance with this hypothesis, the expression of AT1aRs was primarily enhanced in the CA1 microglia of HF rats, which strongly suggests that Ang II could be critical in promoting their pro-inflammatory transition [65]. Moreover, a local increase in Ang II levels has long been known to suppress hippocampal LTP [67,68,69]. Notably, the infusion of losartan, a specific AT1aR antagonist, ameliorated hippocampal inflammation and strongly reduced hippocampal apoptosis [65], thereby rescuing the ability of CA1 pyramidal neurons to properly process the incoming information.

While the secretion of inflammatory cytokines is significantly increased, a recent investigation carried out on a rat model of chronic HF showed that the hippocampal levels of brain-derived neurotrophic factor (BDNF) are decreased [70]. BDNF is indispensable to neurotransmitter release and synaptic plasticity in central synapses [71]. BDNF binds to the tyrosine kinase tropomyosin-related kinase B receptor (TrkB) to potentiate neurotransmitter (glutamate and γ-aminobutyric or GABA) release, to induce and maintain LTP, and to participate in memory consolidation and cognitive function [71,72,73]. Consistent with these notions, BDNF in the cerebrospinal fluid was associated with a severe synaptic loss and impaired synaptic ultrastructure in the hippocampal CA1 region. In addition, chronic HF rats showed a reduction in spatial memory and a down-regulation of the cAMP/PKA/cAMP Response Element-Binding Protein (CREB) pathway, which is crucial for LTP consolidation and memory formation (Figure 2) [70]. Consistent with these findings, Parent and colleagues confirmed that the working spatial memory and the emotional long-term memory are disrupted in the hippocampus and PFC of a rat model of HFrEF due to the down-regulation of 84 genes critical for synaptic plasticity, including those encoding for NMDARs and BDNF [74]. These findings suggest that BDNF deficiency secondary to HF contributes to cognitive decline by impairing glutamatergic synapses and interfering with NMDAR signaling. Intriguingly, the pharmacological blockade of phosphodiesterase-4 (PDE4), which catalyzes cAMP hydrolysis, with Rolipram, BPN14770, or MK0952 has been proposed as a therapeutic approach to rescue cognitive functions in HF [70]. A parallel investigation further showed that the down-regulation of the BDNF/TrkB signaling pathway caused a reduction in glutamate and GABA levels in the brains of HF rats [75]. Intriguingly, a shift in the excitatory/inhibitory (E/I) balance may occur in hypothalamic magnocellular neurosecretory cells (MNCs) of HF rats due to a shift in glutamate–GABA ratio toward a relatively stronger glutamate weight [76], thereby supporting the increased neurohumoral drive in HF [77]. The imbalance of the E/I ratio is involved in the cognitive and emotional deficits described in the majority of CNS pathologies, including neurodegenerative diseases and autism spectrum disorders [22,46,47,78,79,80]. Therefore, future work might assess whether the E/I ratio is affected by HF in brain regions that are involved in emotion and cognition, such as the hippocampus, PFC, amygdala, and cerebellum. Overall, the available evidence suggests that HF may promote cognitive decline by stimulating neuroinflammation, by causing an increase in Ang II levels, and by down-regulating BDNF expression.

## 4. Metabolic Syndrome and Cognitive Impairment

Metabolic syndrome consists of a set of at least five cardio-metabolic disorders that include hypertension, central obesity, hyperglycemia, insulin resistance (IR), and atherogenic dyslipidemia, which may in turn increase the risk of developing type 2 diabetes mellitus (T2DM) and atherosclerosis [81,82,83]. It has been estimated that about one quarter of the world population, i.e., over a billion individuals, is now affected by metabolic syndrome [84], with an increasing prevalence among young individuals due to the larger spread of the Western diet and lifestyle [85]. Individuals with metabolic syndrome are also at strong risk of developing severe neurological deficits, such as cognitive impairment and memory loss, and neuropsychiatric disorders, such as anxiety and depression [23,81,82,86]. The emerging correlation between metabolic syndrome and cognitive impairment could be explained by the subtle reduction in microvascular perfusion that is caused by cerebrovascular atherosclerosis, which results in white matter damage and significantly reduces blood supply to firing neurons [3,87]. This hypothesis is supported by the evidence that subjects suffering from atherosclerosis or T2DM may also be affected by vascular dementia or Alzheimer’s disease [23,86,87]. However, growing evidence suggests that cardio-metabolic alterations may also lead to changes in the neural circuits and molecular mechanisms that underlie memory formation and emotion processing.

### 4.1. Hypertension

Hypertension is regarded as an independent risk factor for Alzheimer’s diseases and vascular cognitive impairment (VCI), i.e., the prodromal stage of cognitive decline that precedes vascular dementia (VD) [88,89,90]. An intensive lowering regimen of mean blood pressure may delay the onset of cognitive deterioration or even preserve cognition in hypertensive subjects [88]. One of the primary mechanisms by which hypertension leads to cognitive impairment is through endothelial dysfunction and microvascular rarefaction [88,89,91], which impair neurovascular coupling (NVC), i.e., the mechanism by which the increased metabolic demand of active neurons is met by an increase in local CBF [59,92]. In addition, hypertension may affect the structural and functional integrity of the BBB, promote microglia activation, and induce an inflammatory response in the brain parenchyma [88,89,91]. Hypertension-induced damage of cerebral microcirculation may obviously exert adverse effects on neuronal activity and synaptic plasticity, but emerging evidence suggests that the onset of cognitive decline in hypertensive individuals is also driven by more subtle molecular alterations.

An early investigation demonstrated an inverse relationship between mean blood pressure and glutamate concentration in the hippocampus of the hypertensive human subjects [93]. Moreover, both the early and late phase of LTP were impaired in the dentate gyrus of a genetically hypertensive rat that was deficient of the nerve growth factor (NGF). Hippocampal expression of TrkB protein was also down-regulated in hypertensive rats [94,95]. Intriguingly, the intracerebroventricular injection of NGF rescued LTP induction and maintenance [94], thereby suggesting that hypertension-induced synaptic impairment may also be due to the down-regulation of neuroprotective growth factors. A follow-up study confirmed that LTP impairment in the dentate gyrus was associated with the disruption of long-term recognition memory and a decrease in BDNF expression [96], as reported above for HF (seeSection 3). The impairment of hippocampal LTP has also been documented in a mouse model of Ang II-induced hypertension [69,97]. In addition, Tucsek and colleagues reported that hypertension was associated with a reduced synaptic density in the mouse hippocampus and with the down-regulation of several genes that are neuroprotective, such as *BDNF* and *Igf1*, or regulate postsynaptic signal transduction events, such as *Homer1* [69]. For instance, insulin-like growth factor-1 (IGF-1) is critical for learning and memory via controlling the induction of NMDARs-dependent Hebbian LTP [98,99], whereas Homer1 mediates metabotropic glutamate receptors-dependent gene expression, which is indispensable for LTP consolidation [100,101]. Consistent with this finding, the administration of IGF-1 has long been known to attenuate the age-dependent decrease in learning and synaptic plasticity [102,103]. In addition, Dai and colleagues demonstrated that the p38 MAPK, which is strongly activated by inflammatory cytokines and thereby supports neuroinflammation [104], is up-regulated in the mouse hippocampus of Ang II-dependent hypertensive mice [97]. The pharmacological blockade of p38 MAPK with the selective inhibitor SKF86002 proved to be effective at rescuing hippocampal LTP induction and, therefore, might provide a suitable strategy for attenuating cognitive decline in hypertension [97]. LTP induction and retention of spatial memory are also impaired in the hippocampus of spontaneously hypertensive rats (SHRs) [105,106,107], which were originally bred from the progenitor Wistar Kyoto Rats. Interestingly, SHRs represent the most widespread animal model of Attention-Deficit/Hyperactivity Disorder (ADHD), which is characterized by a severe deficit in neuropsychological and psychosocial functions, including hyperactivity, inattention, and impulsivity [108,109]. The impairment of synaptic plasticity in SHRs may be due to the increase in oxidative stress [107], the down-regulation of NMDARs [105], CaMKII [110], dopamine D5 receptors [106], and BDNF [111], and the up-regulation of the endosomal Na^+^/H^+^ exchanger member 9 (NHE9) [112,113]. The latter can impair the postsynaptic trafficking of AMPARs [114] and deregulate dendritic Ca^2+^ signaling [113,115,116] during LTP induction. Pre-clinical studies showed that the most effective strategies to rescue synaptic plasticity and spatial learning in SHRs are physical exercise [107,117] and chronic swimming [118]. It should be noted that, unlike the SHR model [105], the hippocampal expression of NMDARs is not affected by Ang II-induced hypertension [69]. As hypertension is a multifactorial disease [119], the molecular mechanisms that favor the cognitive decline are likely to subtly change depending on the underlying etiology. Our current knowledge suggests that hypertension may favor synaptic impairment by reducing glutamate concentration, down-regulating the expression of neuroprotective genes, such as *BDNF* and *Igf-1*, up-regulating Ang II levels and oxidative stress, and interfering with several signaling pathways that support synaptic plasticity, such as NMDARs, AMPARs, and CaMKII.

### 4.2. Obesity

The global obesity epidemic has emerged over the last half century, thereby leading to the widespread diffusion of many chronic diseases that affect the population in the 21st century, including T2DM, insulin resistance, and obesity-related malignancies [120,121]. Many aspects of our contemporary environment may contribute to weight gain, including, but not limited to, sleep deprivation and stress, technology, and the high-caloric Western diet, which is based upon the massive consumption of refined sugars and saturated fat [121,122]. Recent studies revealed a bidirectional relationship between overweight and cognitive functions [123]. While it has long been known that obesity may alter synaptic processes [124,125], detrimental food intake habits may be a consequence of the dysregulation of specific neuronal circuits that are involved in appetite regulation [123,126]. On the other hand, obesity may cause a poorer cognitive performance by impairing learning and memory functions, including episodic memory and working memory [120,127]. The hippocampus has been shown to be rather vulnerable to the metabolic dysfunctions associated with obesity [124,125]. Accordingly, hippocampal-dependent spatial learning and memory are severely affected by a high-caloric diet [128,129,130]. Therefore, obesity is regarded as a novel predisposing risk factor for cognitive disorders, including Alzheimer’s disease and dementia [131,132]. The primary mechanisms by which obesity may lead to cognitive decline include down-regulation of BDNF/TrkA signaling, increased neuro-inflammation and oxidative stress, neurovascular uncoupling, and weakened BBB integrity [127,132,133,134]. However, obesity may also alter the neural circuits and molecular mechanisms that shape spatial learning and memory. 

Early work showed that a high-fat diet impaired both LTP and LTD at the Schaffer collateral-CA1 synapse in mice by impairing glutamate metabolism and down-regulating NMDAR expression [135,136]. A more recent investigation revealed that NF-E2-related factor 2 (Nrf2) deficiency contributes to LTP dysfunction at the CA1 region of mice exposed to a high-caloric diet [137]. In accordance with this, Nrf2 is a transcription factor that is critical to mounting an antioxidant response [138,139] and to preventing cognitive decline with aging [140]. Intriguingly, obesity further exacerbated aging-induced cognitive decline and LTP impairment at the Schaffer collateral-CA1 synapse by promoting the degradation of vasorelaxing and pro-LTP epoxy-eicosatrienoic acids (EETs), such as 8,9-EET and 11,12-EET, and by decreasing the expression of several genes involved in memory formation and storage [141]. Similarly, LTP was decreased at the dentate gyrus of high-fat-fed rats due to the inhibition of group II metabotropic glutamate receptors (mGluR2/3) [142], although neurogenesis was not affected [130]. Conversely, LTP was only reduced in the CA1 region, but not in the dentate gyrus, of Obese Zucker Rats due to calcineurin down-regulation in the latter region that maintains adequate levels of phospho-CaMKII [143]. On the other hand, monosodium glutamate-induced obese mice, which are featured by glucose intolerance, showed enhanced LTP and LTD, as well as impaired excitatory neurotransmission, in the CA1 region due to the up-regulation of the vesicular glutamate transporter 1 [144,145]. Nevertheless, the increased hippocampal excitability led to significant recognition memory deficits, as highlighted by the novel object recognition test [144]. Obesity is also a multifactorial disease [121] and, therefore, it may differentially affect cognitive functions depending on the underlying etiology and the brain region. In this view, it is noteworthy that a high-fat diet was found to abolish LTP in the CA1 region and enhance LTP in the basolateral amygdala [146], which is consistent with the impairment of amygdala-dependent emotional memory reported in both humans and high-fat-fed rats [86,147,148]. Our current knowledge suggests that obesity may promote cognitive decline by interfering with glutamate-mediated neurotransmission, which could be either decreased or enhanced, and impairing the Nrf-2 dependent anti-oxidant response.

A variety of pharmacological strategies have been described aiming to rescue hippocampal LTP induction and restore cognitive functions in obesity, including the following: catecholaminergic stimulation [149]; administration of Glucagon-like peptide-1 (GLP-1) agonists, such as exendin-4 [150], or the insulin sensitizer, metformin [151]; blocking IL-1 signaling with the IL-1 receptor antagonist (IL-1RA) [152]; and rescuing the E/I ratio with a mixture of memantine and allopregnanolone, which, respectively, block NMDARs and GABA_A_ receptors. These studies strongly suggest that the molecular mechanisms responsible for obesity-induced cognitive decline include the alteration of these signaling pathways. However, the multifactorial nature of obesity is unlikely to benefit of a generalized approach, e.g., supplementation of diets enriched with curcumin and omega-3 [122] or Mediterranean diet [82], but rather requires an individualized approach to effectively manage obesity according to its causation [121].

### 4.3. Hyperglycemia and Insulin Resistance

Long-term chronic hyperglycemia can be due to a deficiency in insulin production or the development of insulin resistance, which can result in type 1 diabetes mellitus (T1DM) or T2DM [153]. Both types of diabetes may cause mild to moderate cognitive impairment, a significant pathological condition known as diabetic encephalopathy [154]. Furthermore, diabetes mellitus is regarded as a risk factor for Alzheimer’s disease [155,156]. Endothelial dysfunction is critical in mediating the impairment of cognitive decline by hyperglycemia/diabetes due to the disruption of the BBB [157] and neurovascular uncoupling [158]. Studies carried out in rodent models of hyperglycemia, T1DM, and T2DM also suggested that oxidative stress and neuroinflammation may cause cognitive impairment by damaging myelinated tracts, hippocampal neuronal circuits, and synaptic contacts [159,160,161,162]. Furthermore, electrophysiological abnormalities, including alterations in glutamatergic transmission throughout the CNS and in hippocampal-dependent learning, memory, and cognitive tasks, have been reported [163,164,165].

By using the streptozotocin-induced rat model of T1DM, it has been shown that high-frequency stimulation (HFS) of the Schaffer collateral only results in weak LTP induction, if any, while low-frequency stimulation (LFS) induces a larger LTD as compared to healthy animals [166,167,168,169]. In accord, spatial learning and recognition memory were impaired only in severely hyperglycemic rats [166,170,171]. However, LFS results in a weaker LTD in juvenile streptozotocin-induced rats due to the reduction of cholinergic stimulation of the hippocampus [172,173,174]. These findings suggesting the age of diabetes onset could determine whether it also results in cognitive defects or not. Several mechanisms accounted for the impairment of LTP in the hippocampal CA1 region, including a defect in presynaptic glutamate release [155], the down-regulation of NMDAR expression [175] and CaMKII-dependent phosphorylation [176,177], a reduction in the Ca^2+^-dependent recruitment of postsynaptic AMPARs [167] and AMPAR-mediated EPSCs [178], a rightward shift in the threshold of LTP induction, and a leftward shift in the threshold of LTD induction [179]. More recent investigations further showed that T1DM may interfere with synaptic potentiation by decreasing the activity of the Na^+^/K^+^ ATPase [171,180], thereby preventing the restoration of the ionic milieu during sustained neuronal activity [181]. Preliminary evidence indicates that the impairment of LTP induction at the Schaffer collateral-CA1 synapse was associated with a reduction in AMPA/NMDA ratio in young adult, but not juvenile, streptozotocin-injected rats [172]. This finding strongly suggests that the age of onset of T1DM might be considered for selecting the most appropriate therapy to prevent cognitive decline in patients. It should, however, be noted that insulin treatment rescues synaptic potentiation in the hippocampal CA1 region [182,183]. Our current knowledge suggests that T1DM may impair LTP induction by interfering with glutamate release and glutamate-dependent postsynaptic signaling, including the down-regulation of NMDARs, AMPARs, and CaMKII activation, and by preventing the restoration of the ionic gradients across the neuronal membrane after intense synaptic activity upon Na^+^/K^+^ ATPase inhibition.

Therapeutic strategies that improved hippocampal-dependent learning and memory by boosting NMDAR signaling and ameliorating LTP impairment in T1DM include the nerve-protective drug extracted from the seed of Chinese celery, l-3-*n*-Butylphthalide (NBP) [184], the isoquinoline alkaloid berberine [185], the phenolic compound vanillic acid [186], probiotics treatment [187], inhibition of the receptor for advanced glycation end products (RAGE) with the specific blocker FPS-ZM1 [188], physical exercise [189], and stem cell transplantation [190].

Similar results have been obtained in multiple rodent models of T2DM. Impairment of LTP induction in the hippocampal CA1 region of Obese Zuker rats is caused by the alteration of pre-synaptic glutamate release [143,191]. Reduced LTP and impaired spatial learning ability have also been documented in high-glucose-fed rats due to a significant reduction in postsynaptic spine density and BDNF levels [192,193]. Finally, the magnitude of hippocampal LTP was dramatically attenuated in Otsuka Long-Evans Tokushima Fatty (OLETF) rats, which spontaneously develop T2DM [194]. HFS-induced LTP at the Schaffer collateral-CA1 region was also impaired in mouse models of T2DM, including a transgenic murine model of adipocyte insulin resistance (AtENPP1-Tg) [195], diabetic db/db mice [196,197], spontaneous obese KK-Ay type 2 diabetic mice [198], and transgenic mice deficient of insulin receptor β subunit [199] or GLP-1 [200]. The molecular mechanisms involved in T2DM-dependent impairment of LTP and hippocampal-related learning and memory functions include the alteration in the molecular assortment of AMPAR subunit [198], down-regulation of NMDAR expression and phosphorylation [198], reduced glutamate release [200], CaMKII activation [196,201], CREB expression [201] during HFS, and deregulation of GABAergic signaling [197]. The deficiency in hippocampal LTP, as well as the impairment of memory recognition and spatial learning, could be ameliorated by a variety of treatments, including diet intervention [198], NBP [196], RAGE inhibition with FPS-ZM1 [202], stem cell transplantation [195], metformin, and environmental enrichment [193]. Sodium-glucose cotransporter-2 (SGLT2) inhibitors, which have been developed as anti-diabetic drugs, also proved to be more effective at tempering cognitive dysfunction as compared to other anti-diabetic strategies [203,204,205,206]. Preliminary evidence showed that SGLT2 inhibitors could rescue synaptic plasticity by increasing the hippocampal levels of BDNF and NGF [204] and that dapagliflozin was able to improve LTP at the Schaffer collateral-CA1 synapse [207]. Due to the emergence of SGLT2 inhibitors as pleiotropic drugs that exert a broad range of beneficious systemic effects, future investigations should assess whether and how they directly affect pre- or postsynaptic mechanisms of excitatory and inhibitory neurotransmission.

### 4.4. Dysregulated Endothelial Ion Signaling 

As anticipated above, endothelial dysfunction is involved in metabolic syndrome-induced cognitive decline by mining the BBB integrity, by supporting arterial stiffening and microvascular rarefaction, and by favoring neurovascular uncoupling. Recent investigations showed that cerebrovascular endothelial cells may sense synaptic activity and thereby release nitric oxide (NO) to increase blood supply to firing neurons [59,92,208]. Synaptically released neurotransmitters and neuromodulators, such as glutamate, may induce endothelial Ca^2+^ oscillations at the postarteriolar transition zone by activating G_q_-protein-coupled receptors that lead to Ca^2+^ release from the endoplasmic reticulum through inositol-1,4,5-trisphosphate receptors [116,209,210]. This oscillatory increase in endothelial Ca^2+^ concentration can be enhanced by endothelial hyperpolarization through inward rectifier K^+^ channels (K_IR_2.1) [209,211] and stimulates the endothelial NO synthase (eNOS), thereby leading to NO-dependent vasorelaxation and NVC [116,209,212]. In addition, cerebrovascular endothelial cells express NMDARs that can also be activated by synaptic activity and are physically coupled to eNOS and NO production [25,213,214,215]. The metabolic syndrome may compromise the endothelial ion signaling machinery [92,134,216,217,218,219,220,221], and a growing body of evidence shows that endothelial K_IR_2.1 channels [222,223,224] and NMDARs [213] may be impaired in Alzheimer’s disease [225]. This would, in turn, lead to reduced eNOS activation and impaired NO signaling at the neurovascular unit. Intriguingly, eNOS-derived NO may also regulate LTP induction and maintenance in the hippocampal CA1 region [226,227,228,229], and the LTP/LTD balance is shifted towards LTD upon the genetic deletion of eNOS [230]. Endothelial ion signaling can be targeted by pharmacological manipulation and dietary interventions [92,134,229]. Therefore, future work should assess whether the metabolic syndrome also affects endothelial ion signaling at the neurovascular unit and whether this contributes to the defects observed in hippocampal-dependent learning and memory cognitive tasks.

## 5. Arrhythmias

Cardiac arrhythmias, including atrial fibrillation, ventricular tachycardia, and ventricular fibrillation, may cause cognitive decline and dementia through a variety of mechanisms, such as cerebral hypoperfusion, thromboembolism, inflammation, and stroke [231,232,233]. Stroke is primarily caused by atrial fibrillation and is known to cause a maximum reduction in cognitive function [234], whereas anoxic brain injury is more commonly associated with ventricular arrhythmias [235]. Untangling the molecular mechanisms linking cardiac arrhythmias to the impairment of the synaptic mechanisms underlying memory formation and storage requires the use of appropriate transgenic animal models. But fatal arrhythmic events could interfere with the successful exploitation of electrophysiological experiments or voltage-sensitive dye imaging in ex vivo slices and in vivo brain preparations. For instance, several mouse models of spontaneous atrial fibrillation have been developed, but a straightforward analysis of LTP induction and maintenance, e.g., in the hippocampal CA1 region, is still missing [236]. Notably, myocardial fibrosis, which is the primary adaptative response to myocardial infarction, may also predispose HF patients to ventricular tachycardia and ventricular fibrillation [237]. Therefore, we cannot rule out that, in these patients, cognitive decline is driven by HF rather than by cardiac arrhythmias. An intriguing possibility is that inheritable arrhythmias underlain by mutations in ion channels that are expressed in both the heart and the brain are also associated with cognitive impairment. Mutations in type 2 ryanodine receptors (RyR2) may lead to cardiac arrhythmias, such as catecholaminergic polymorphic ventricular tachycardia and arrhythmogenic cardiomyopathy [238,239,240], and to cognitive dysfunctions, such as Alzheimer’s disease [241]. However, mutations in the *RYR2* gene cause either cardiac or neurological disorders, not both. Conversely, a genetic mouse model of the Timothy syndrome, a multi-organ form of long QT syndrome that is caused by mutations of the *CACNA1C* gene [238,242], has recently been exploited to investigate the molecular mechanisms of the neurological deficits, such as autism spectrum disorders, associated with this rare cardiac disease. In accord, the *CACNA1C* gene encodes for the voltage-dependent L-type Ca^2+^ channel, Ca_V_1.2, which regulates the excitation–contraction coupling mechanism in the heart [243] and Ca^2+^-dependent gene expression and synaptic plasticity in the brain [244]. A sporadic single nucleotide change, which produces a missense mutation (G406R) in the pore-forming subunit of Ca_V_1.2, is the most common cause of the Timothy syndrome [242]. This mutation exerts a gain-of-function effect that shifts the threshold of activation towards more negative potentials and removes the voltage-dependent inactivation of L-type Ca^2+^ currents, thereby leading to intracellular Ca^2+^ overload [242]. A preliminary investigation confirmed that the intracellular Ca^2+^ signals produced by Ca_V_1.2 G406R channels showed larger spatial spread and amplitude as compared to wild-type channels. However, the duration of the increase in intracellular Ca^2+^ concentration was significantly shorter, thus suggesting the involvement of negative-feedback mechanisms that limit Ca^2+^ entry through Ca_V_1.2 G406R channels in neurons [245]. It has been suggested that the cellular outcome of the G406R mutation in the CNS could be slightly different than in the heart due to splice variants and the interaction with other ancillary subunits and regulatory proteins [246]. A subsequent report showed that the mutant Ca_V_1.2 G406R channels drive CREB-dependent gene expression in a depolarization-independent manner due to the channel activation at sub-threshold potentials [247]. Intriguingly, the occupancy of the Ca^2+^-binding site within the selectivity filter, but not Ca^2+^ entry into the cytoplasm, is required for the basal transcriptional activity of Ca_V_1.2 G406R channels [247]. This finding is consistent with the emerging view that both ionotropic receptors and voltage-gated channels, including Ca_V_1.2, may signal in a flux-independent manner [25,248,249]. A recent investigation explored the impact of the G406R mutation in hippocampal synaptic plasticity by exploiting a novel transgenic mouse model, in which the expression of the Ca_V_1.2 G406R mutant protein from exon 8 was blunted via transcriptional interference to prevent fatal cardiac arrhythmias [246]. Ca^2+^ entry through L-type Ca_V_1.2 channels during plasticity induction may support the NMDARs-mediated recruitment of Ca^2+^-dependent signaling pathways that stimulate AMPARs and GABA_A_ receptor trafficking and postsynaptic spine remodeling and expansion [18,22,250,251,252]. This novel transgenic mouse model showed that the E/I balance was shifted towards excitation in the CA1 region due to an increase in AMPARs-mediated excitatory transmission and a decrease in GABA_A_ receptors-mediated inhibitory transmission [246]. The loss of GABAergic inhibition could also be due to a reduction in interneuron migration, which is also driven by Ca^2+^ influx through Ca_V_1.2 channels [253]. That the *CACNA1* gene could provide a molecular link between inherited arrhythmias and cognitive dysfunction is also suggested by the deletion of exon 33, which causes a gain-of-function mutation in the Ca_V_1.2 protein. The absence or decrease in exon 33-containing Ca_V_1.2 channels results in ventricular tachycardia and lengthened QT interval [254], as well as in severe neurological deficits [255]. In accord, the deletion of exon 33 caused an increase in late LTP and favored the transition of early LTP to long-lasting LTP at the Schaffer collateral-CA1 synapse. Furthermore, LFS did not induce LTD but rather synaptic potentiation [255]. The LTP/LTD imbalance did not improve hippocampal-dependent functions, such as associative memory, while disrupting social behaviors as they became less aggressive [255]. In accordance with this evidence, the *CACNA1* gene is also widely expressed in the amygdala [244], but the electrophysiological evidence that synaptic mechanisms are altered by Ca_V_1.2 mutation in this brain region is missing. Future work is required to assess whether the deletion of exon 33 from the *CACNA1* gene has any pathological relevance for human health.

## 6. Conclusions

Cardiogenic dementia is a detrimental consequence of HBA that can exacerbate the progression of CV disorders and worsen the prognosis and management of the patients. Cardiogenic dementia can increase mortality, reduce the quality of life, and enhance the economic burden imposed on the families of CV patients. The framework of the pathophysiological mechanisms by which disruption of the HBA leads to cognitive impairment, i.e., cerebral hypoperfusion, neuroinflammation, BBB breakdown, microvascular rarefaction, and neurovascular uncoupling, has roughly been delineated. But obtaining novel insights into the subtle cellular and molecular mechanisms that underpin memory storage and formation, as well social behavior and emotional responses, requires a strong interaction between cardiovascular physiologists and molecular neurobiologists. Most of the attention has hitherto been paid to the CA1 region, which is critical to the formation, consolidation, and retrieval of hippocampal-dependent memories. The available evidence suggests that CV disorders tend to converge on the dysregulation of the same synaptic mechanisms, e.g., glutamate release, AMPARs, NMDARs, and CaMKII activation, to reduce the antioxidant defenses and to down-regulate neuroprotective genes, such as *BDNF* and *Igf-1*. In our opinion, future studies will have to do the following: (1) consolidate these preliminary findings, not only in the CA1 region but also in the other hippocampal areas; (2) gain further insights on the alterations in the E/I balance that could favor LTD over LTP and thereby boost the cognitive decline; (3) confirm that endothelial signaling plays a critical role in cognitive tasks, as also recently shown in [256], and assess the contribution of endothelial dysfunction to cognitive impairment not only in terms of cerebral hypoperfusion but also of direct regulation of neuronal activity and synaptic transmission [25,229]; (4) investigate whether the age of onset of the CV disorder, e.g., obesity and diabetes, may affect the extent and the mode of cognitive dysfunction; and (5) exploit induced pluripotent stem cell-derived neuronal cultures generated from CV patients to boost the therapeutic translation of animal studies. As recently outlined by an American Heart Association scientific statement [257], there is still a gap between our understanding of how CV diseases impact on brain function at macroscopic (e.g., cerebral hypoperfusion and cerebral microembolism) and microscopic (e.g., synaptic transmission and LTP induction) levels. 

We expect that future work will have to focus on other brain regions, such as the PFC, amygdala, and cerebellum, that also play a crucial role in various cognitive and behavioral functions. For instance, PFC degeneration has been shown to correlate with cognitive decline in older adults [258]. The PFC function is seemingly insensitive to changes in the heart rate [259]. However, recent studies provided evidence that children and adolescents with congenital heart disease showed lower cognitive performance, mainly in episodic memory, executive function, and language, which are associated with cerebello–PFC connectivity [260,261]. Furthermore, the structure and/or the activity of PFC, amygdala, and cerebellum may also be affected by HF [262,263,264], insulin resistance [265], overweight and obesity [266,267,268], and atrial fibrillation [269,270,271]. Functional magnetic resonance imaging has clearly demonstrated that the different brain areas that underlie cognitive, executive, and emotional functions are intimately interconnected and effectively interact to shape human behavior. Therefore, clinical studies are expected to unveil how CV disorders affect the resting-state functional connectivity and the coordination of these interactions, whereas basic research will have to exploit animal models of such diseases to dissect the underlying alterations at cellular and molecular levels.

The advent of novel, high-resolution recording tools, such as high-density multielectrode arrays and multiphoton imaging combined with rapid voltage-sensitive dye imaging, is predicted to offer novel insights on the alterations of neural circuits and synaptic mechanisms, both ex vivo and in vivo. The design of more appropriate therapeutic strategies to delay or prevent cardiogenic dementia will also benefit from optogenetics, which is yet to be applied to investigate the synaptic mechanisms of cognitive decline in mouse models of CV diseases. 

## Data Availability

No new data was created for this manuscript.
